# Dual diagnosis – dual intervention: a complex challenge in contemporary mental health care

**DOI:** 10.1192/j.eurpsy.2026.10324

**Published:** 2026-07-31

**Authors:** J. Samochowiec

**Affiliations:** Department of Psychiatry, Pomeranian Medical University, Szczecin, Poland

## Abstract

**Abstract:**

The management of patients with schizophrenia co-occurring with substance use disorders (dual diagnosis) represents one of the most complex challenges in contemporary psychiatry and requires an integrated therapeutic approach addressing both psychotic symptoms and addictive behaviors simultaneously. Sequential treatment models, in which substance use is managed prior to the psychiatric disorder, are increasingly considered insufficient and are associated with a higher risk of relapse, rehospitalization, and deterioration of social functioning.

Patients with dual diagnosis are characterized by greater variability in the course of psychosis, increased vulnerability to symptom exacerbation following substance exposure, poorer response to treatment, higher clinical instability, and an elevated risk of extrapyramidal symptoms and adverse effects related to polypharmacy. Current clinical recommendations emphasize the preferential use of antipsychotics with a low metabolic and neurological burden, avoidance of sedating agents and combinations that potentiate central nervous system depression, careful assessment of pharmacokinetic interactions (particularly involving CYP3A4 and CYP2D6 pathways), and consideration of long-acting injectable formulations in patients with low adherence.

Despite the high prevalence of dual diagnosis, most clinical guidelines remain disorder-specific and provide limited practical guidance on antipsychotic selection in this population. In this clinical gap, therapeutic decisions should be guided by pharmacodynamic profiles, impact on reward circuitry, and real-world clinical evidence. Emerging expert literature increasingly supports the use of D2/D3 partial agonists, particularly agents with high D3 affinity, as a mechanistically coherent strategy aligned with the neurobiology of both psychosis and addiction.

Importantly, effective management must also include systematic identification and treatment of somatic complications related to substance use and antipsychotic therapy, as untreated physical comorbidities significantly worsen prognosis, adherence, and overall clinical outcomes.

**Disclosure of Interest:**

None Declared

